# Application and optimization of prostate-specific antigen screening strategy in the diagnosis of prostate cancer: a systematic review

**DOI:** 10.3389/fonc.2023.1320681

**Published:** 2024-01-09

**Authors:** Zhengchao Zhang, Aimin Tian, Jizhong Che, Yandong Miao, Yuanyuan Liu, Yangyang Liu, Yankai Xu

**Affiliations:** ^1^ Department of Urology, Yantai Affiliated Hospital of Binzhou Medical University, The Second Clinical Medical College of Binzhou Medical University, Yantai, Shandong, China; ^2^ Department of Oncology, Yantai Affiliated Hospital of Binzhou Medical University, The Second Clinical Medical College of Binzhou Medical University, Yantai, Shandong, China

**Keywords:** prostate cancer, PSA screening, prognosis, mortality, overdiagnosis

## Abstract

Currently, prostate cancer (PCa) poses a global risk to the well-being of males. Over the past few years, the utilization of prostate-specific antigen (PSA) screening has become prevalent in the identification and management of PCa, which has promoted a large number of patients with advanced PCa to receive timely treatment and reduce the mortality. Nevertheless, the utilization of PSA in PCa screening has sparked debate, and certain research has validated the potential for overdiagnosis and overtreatment associated with PSA screening. Hence, in order to decrease the mortality rate of PCa patients and prevent unnecessary diagnosis and treatment, it is crucial to carefully choose the suitable population and strategy for PSA screening in PCa. In this systematic review, the clinical studies on PSA screening for the diagnosis and treatment of PCa were thoroughly examined. The review also delved into the effects and mechanisms of PSA screening on the prognosis of PCa patients, examined the factors contributing to overdiagnosis and overtreatment, and put forth strategies for optimization. The objective of this research is to offer valuable recommendations regarding the utilization of PSA screening for the detection and management of PCa.

## 1 Introduction

At present, prostate cancer (PCa) ranks as the second leading factor for male cancer fatalities. By the year 2020, it is estimated that there will be around 1.4 million fresh instances and 375,000 fatalities globally. Furthermore, PCa stands as the prevailing form of cancer among males in over half of the nations across the globe (1). The occurrence and mortality rates differ depending on the geographical area, where African males face a high morbidity 26.6 age-standardized rate per 100 000 (ASR) and mortality 14.6 ASR, while Asian males face a decreased morbidity 11.5 ASR and mortality 4.5 ASR (2). Over the past few years, one study reported that PCa screening can reduce the death rate of prostate cancer by 20% (3). Therefore, PSA screening has made great progress in the diagnosis of PCa because of its importance.

**Figure 1:**
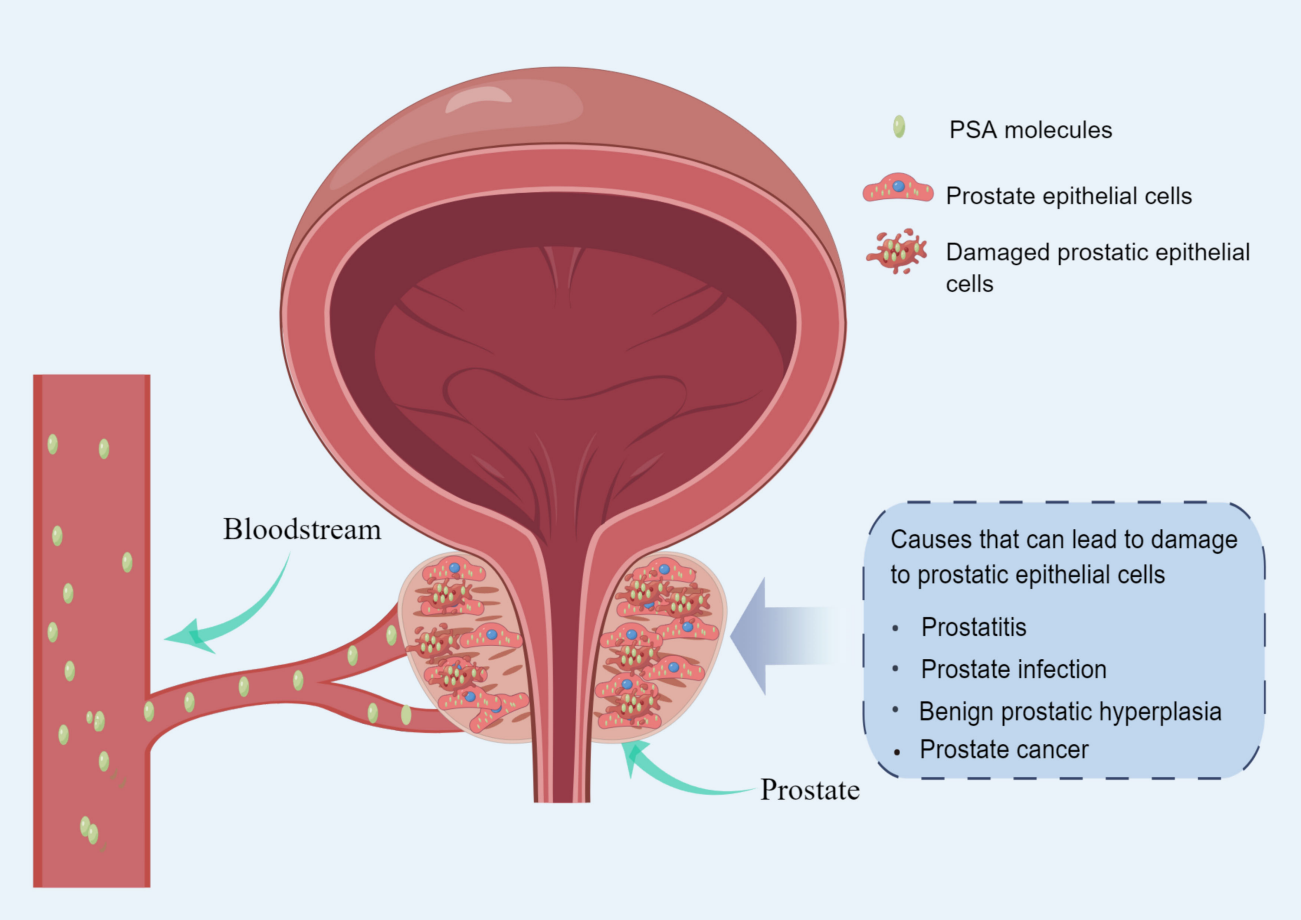
Diagram of the mechanism of PSA release into the blood circulation (made by online Figdraw).

PCa screening is a medical diagnostic practice based on prostate-specific antigen (PSA) testing. PSA is an enzyme produced by the prostate that degrades a gelatinous semen protein, thereby releasing motile sperm (4, 5). When prostate epithelial cells are destroyed by tumors, large amounts of PSA are released into the bloodstream (5). PSA levels are also elevated when the prostate is inflamed, infected, or benign prostate hyperplasia (6, 7) (**Figure 1**). As a result, Elevated PSA is not enough to make a definitive diagnosis of prostate cancer (8). However, PSA can screen out potential PCa patients from the population who need further diagnosis (9). PSA testing is commonly used in middle-aged and older men with lower urinary tract symptoms (LUTS) and in asymptomatic men at risk for PCa (10). Patients with elevated PSA often require prostate magnetic resonance imaging (MRI) and/or prostate biopsy for further diagnosis (11).

The PSA testing was approved by the U.S. Food and Drug Administration (FDA) in 1986 for the purpose of monitoring the advancement of PCa. In 1994, the FDA approved PSA for PCa screening in asymptomatic men. Consequently, there was a significant increase in the prevalence of PCa during the 1980s and 1990s, primarily attributed to the extensive utilization of PSA screening (12). PCa screening is aimed at asymptomatic men. The significance of PCa screening is to decrease the death rate of PCa in the screened population while maintaining the quality of life for those being screened (13). The primary purpose of PCa screening is to enhance the rate of identifying PCa and identifying PCa at an early stage, particularly PCa that is clinically significant. Men who are in good physical condition and have a life expectancy exceeding 10 years should undergo PSA-based PCa screening every two years, constituting the current focus of the screening target population for PCa. It is important to focus on PCa screening among high-risk populations, which include males aged over 50, males aged over 45 with a familial background of PCa, and males aged over 45 with BRCA2 gene mutations (14). This is particularly important for developing population screening strategies for PCa.

While the mortality of PCa has been decreased by PSA screening (3), research has indicated that 20 to 60 percent of cancers identified through PSA testing are instances of overdiagnosis (15, 16). The long-term fatal risk of PCa remains very low, especially in developed countries (17, 18). Currently, there exist notable variations in the prevalence and fatality rates of PCa across nations. Approximately 81% of newly reported cases in the United States are classified as clinically localized PCa, whereas the percentage is only 33% in China. The remaining cases consist of advanced or metastatic patients (19). The extensive PSA screening in Europe and the United States is likely responsible for these findings. The prevalence of PCa in the United States increased significantly starting in the late 1980s due to the widespread adoption of PSA-based screening (20). Consequently, implementing PSA screening for high-risk populations in developing nations is a crucial approach for the early detection and management of clinically significant PCa. In 2012, the US Preventive Services Task Force (USPSTF) objected to PSA-based PCa screening, stating that the drawbacks of screening outweigh the advantages with reasonable confidence.This review presents a comprehensive examination of researches on the utilization of PSA screening for PCa. The discussion focused on the importance of its value in diagnosing and treating PCa. Besides, an examination was conducted on the factors contributing to the excessive treatment of PCa. Related studies ultimately presented the optimization plan for PSA screening in detecting PCa. The aim is to provide valuable suggestions for the optimization of PCa screening strategies.

## 2 Materials and methods

This systematic review adhered to the guidelines of the preferred reporting items for systematic reviews (PRISMA). In order to obtain randomized and non-randomized screening studies on the utilization of PSA screening in PCa diagnosis, we conducted searches in the most pertinent databases (PubMed and Web of Science). Using the Boolean operator, we combined the terms (PCa and PSA screening) OR (PCa screening and PSA). We considered articles that were published in the English language between July 2013 and July 2023.All of the published articles included in this collection were clinical studies that reported on the utilization of PSA screening for the diagnosis of PCa. Reviews, retrospective and cross-sectional studies were not considered. Due to the variation in age, screening techniques, and regional disparities in PCa treatment among the participants in the study, a meta-analysis was not conducted. Two reviewers evaluated the quality of bias in all the studies included, using the Cochrane risk bias assessment tool. The outcome indicators of PSA screening were determined by utilizing clinical prognostic indicators of patients.

## 3 Results

### 3.1 Literature search

**Figure 2:**
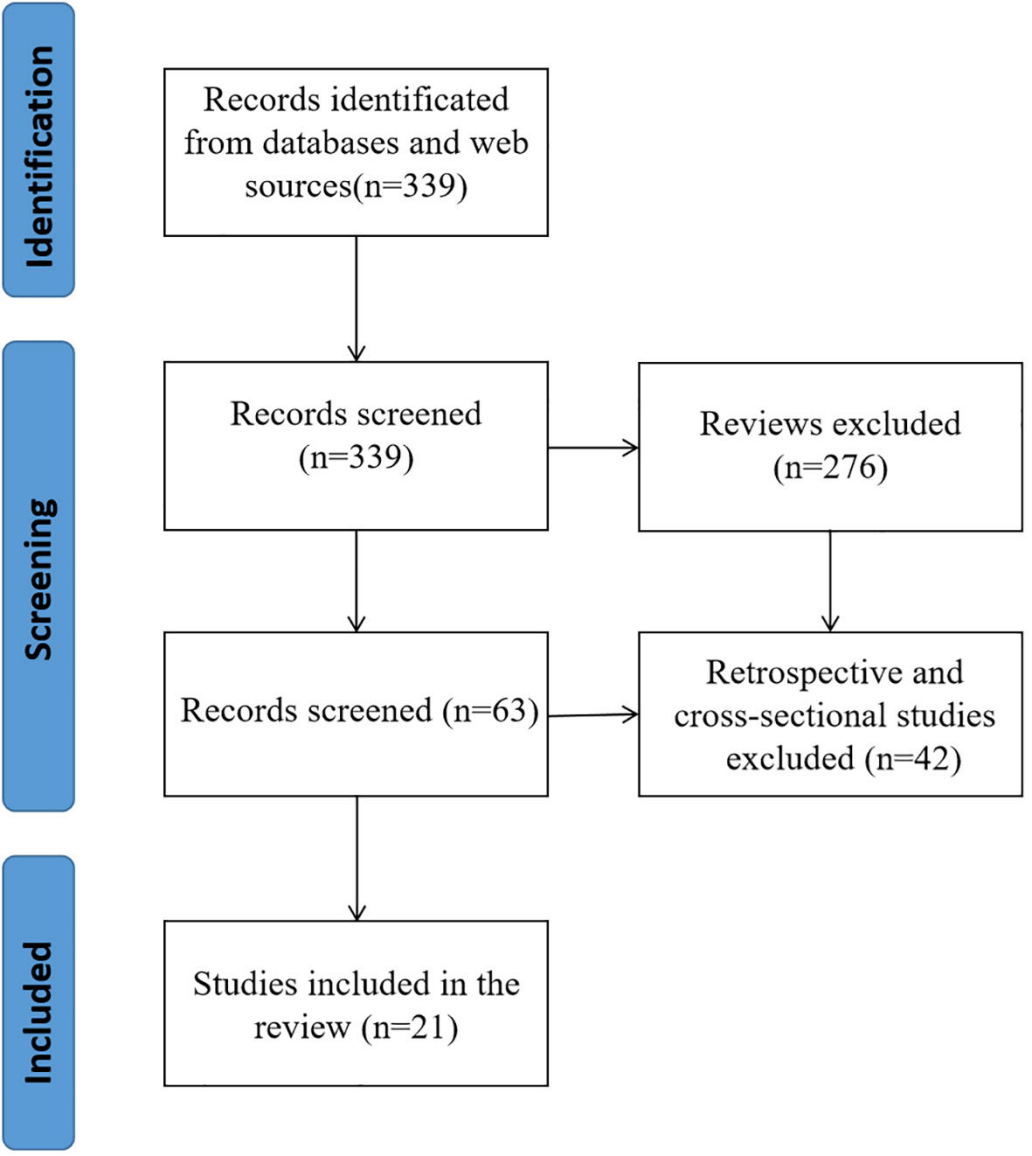
The systematic review flow diagram of selected studies.

PubMed and Web of Science yielded a total of 339 articles through a comprehensive search. The retrieved literatures were then screened, and reviews(276), retrospective studies and cross-sectional studies (21) were excluded. In the end, a total of 21 pieces of literature were incorporated into the ultimate examination (**Figure 2**).

### 3.2 Basic characteristics

The studies included in this review were published between July 2013 and July 2023. Fifteen randomized screening trials and five non-randomized screening trials were included. All studies included people aged 45-85 years. All included studies were conducted in Sweden, Finland, Netherlands, Spain, United States, England, Germany, China, and some European countries. Three of the studies included more than 100,000 people, five more than 50,000 people, and 16 more than 10,000 people (**Table 1**).

**Table 1:** Study characteristics.

References	Published time	Type of Study	Region	Study period	Number of Patients	Age/ranges of ages. (years)
A. Grenabo Bergdahl et al. (22)	2013	Randomized Screening Trial	Sweden	1995-2012	13,423 patients	50 ~ 65
T. P. Kilpeläinen et al. (23)	2013	Randomized Screening Trial	Finland	1996-2010	80,144 patients	55 ~ 67
M. J. Roobol et al. (24)	2013	Randomized Screening Trial	City of Rotterdam, Netherlands	1993-2010	42,376 patients	54 ~ 74
L. P. Bokhorst et al. (25)	2014	Randomized Screening Trial	City of Rotterdam, Netherlands	1993-2010	34,833 patients	55 ~ 69
M. Luján et al. (26)	2014	Randomized Screening Trial	Spanish	1998-2013	4,276 patients	45~ 70
A. J. Vickers et al. (16)	2014	Non-randomized Screening Trial	United States	1987-2013	2,774 patients	45~ 85
R. Arnsrud Godtman et al. (27)	2015	Randomized Screening Trial	Sweden	1995-2012	20,000 patients	67 ~ 71
C. Buzzoni et al. (28)	2015	Randomized Screening Trial	Europe	1998-2014	162,338 patients	60.2
E. A. Heijnsdijk et al. (29)	2015	Non-randomized Screening Trial	Sweden	1995-2012	46,000 patients	55 ~ 69
M. Luján et al. (30)	2015	Randomized Screening Trial	Spanish	1998-2014	4,276 patients	45 ~ 70
S. Carlsson et al. (31)	2017	Non-randomized Screening Trial	Sweden	1995-2012	7,539 patients	50 ~ 55
J. Hugosson et al. (32)	2018	Randomized Screening Trial	Sweden	1995-2012	20,000 patients	50 ~ 64
R. M. Martin et al. (33)	2018	Randomized Screening Trial	England	2001-2016	419,582patients	50 ~ 69
J. Hugosson et al. (34)	2019	Non-randomized Screening Trial	Europe	1998-2014	182,160 patients	55 ~ 69
M. Luján Galán et al. (35)	2020	Randomized Screening Trial	Spanish	1998-2019	4,276 patients	45 ~ 70
K. Talala et al. (36)	2020	Randomized Screening Trial	Finland	1996-2020	80,458 patients	55 ~ 67
Z. Zhang (37).	2021	Non-randomized Screening Trial	Chain	2019-2021	13,726 patients	50-80
T. Pakarainen et al. (38)	2021	Randomized Screening Trial	Finland	1996-2015	31,867 patients	55 ~ 67
S. D. Walter et al. (12)	2021	Randomized Screening Trial	Finland	1996-2015	80,458 patients	55 ~ 67
C. Arsov et al. (39)	2021	Non-randomized Screening Trial	Germany	2014-2019	46,642 patients	45 ~ 50
M. Frånlund et al. (40)	2022	Randomized Screening Trial	Sweden	1994-2016	20,000 patients	69

### 3.3 Main findings

#### 3.3.1 Impact of PSA screening on prognosis of patients with PCa

The controversy surrounding the improvement of outcomes for men with PCa through screening persists, as the potential negative effects of excessive testing and unnecessary treatment may surpass the advantages in terms of potential mortality or quality of life (40). Over the past few years, numerous clinical studies have been carried out by researchers, the majority of which have discovered that screening for PCa can greatly decrease patient mortality. Additionally, PSA screening has been found to lower the occurrence of PCa metastasis, and early treatment can be initiated through screening cases (24, 25, 28, 32, 34, 40, 41). Nevertheless, certain studies that have conducted extensive monitoring over a prolonged period have discovered that PSA screening does not provide any advantage in terms of survival (23, 26, 30, 33, 35, 36) (**Table 2**). This is primarily attributed to a decrease in long-term mortality caused by PCa. Therefore, it is particularly important to explore the influencing factors of PSA screening on the prognosis of PCa patients for optimizing PCa screening.

**Table 2:** Summary of relevant studies on the application of screening to the treatment of localized PCa.

References	Follow-up time	Objective	Main findings
A. Grenabo Bergdahl et al. (22)	12 years	The objective was to investigate the risk of PCa in a population after discontinuation of PSA screening	Nine years after PSA screening stopped, the incidence of potentially fatal cancers was comparable to that of men who had not been screened.
T. P. Kilpeläinen et al. (23)	12 years	The study evaluated PCa-specific mortality in a Finnish PSA screening population.	Twelve years of PSA screening resulted in a small reduction in PCa-specific mortality, but the difference was not statistically significant compared with the control group, and the cost was moderate overdiagnosis.
M. J. Roobol et al. (24)	10 years	This study primarily assessed PCa-specific mortality in the Rotterdam portion of the ERSPC trial.	PSA screening reduced prostate-specific mortality by 32% in the 55-69 year age range.
L. P. Bokhorst et al. (25)	13 years	This study examined the reduction in PCa mortality following PSA screening.	PSA screening reduced the risk of death from PCa by 51 percent compared to men who did not undergo PSA screening.
M. Luján et al. (26)	15.2 years	This study presents the long-term follow-up results of PCa screening trials conducted in the Mediterranean region.	This study was not able to demonstrate a benefit of PSA screening in PCa-specific mortality.
A. J. Vickers et al. (16)	25 years	The aim of the study was to assess the impact of age and baseline PSA on overdiagnosis of PSA screening.	Overdiagnosis of PCa is strongly correlated with age and PSA levels.
R. Arnsrud Godtman et al. (27)	18 years	The aim of this study was to compare the ability of organized and opportunistic screening to reduce mortality and the risk of overdiagnosis in PCa.	Organized PSA screening reduces PCa mortality but is associated with overdiagnosis. Opportunistic PSA screening does not reduce mortality from PCa and also leads to overdiagnosis.
C. Buzzoni et al. (28)	13.0 years	The study evaluated the potential impact of PSA screening on PCa mortality.	Patients in the screening group were diagnosed with significantly less metastatic PCa.
E. A. Heijnsdijk et al. (29)	—	This study explores optimizing PSA screening strategies in terms of reducing mortality, overdiagnosis and costs, and improving quality of life.	PSA screening is cost-effective for people aged 55 to 59 years, while PSA screening is less cost-effective for people aged 63 years and older.
M. Luján et al. (30)	15.8 years	The purpose of the study was to prove whether PSA screening reduced PCa mortality.	PCa screening can shift the diagnosis to an earlier stage. However, a benefit in terms of overall survival or cancer-specific survival has not been demonstrated.
S. Carlsson et al. (31)	17 years	The objective of this study was to evaluate the effect of PSA screening on PCa mortality when PSA screening was started at age 50 to 54 years.	PSA screening reduces PCa mortality in men aged 50-54.
J. Hugosson et al. (32)	18 years	This study examined whether PSA screening could reduce PCa mortality over 18 years of follow-up.	PSA screening reduces PCa mortality and may reduce sociodemographic inequalities in PCa mortality.
R. M. Martin et al. (33)	10 years	This study evaluated the impact of PSA screening and standardized diagnostic approaches on PCa -specific mortality.	There was no significant difference in PCa mortality between the two groups after a median follow-up of 10 years, but the detection of low-risk PCa cases increased in the PSA screening group.
J. Hugosson et al. (34)	16 years	The study investigated whether PSA screening could reduce PCa mortality for up to 16 years.	Early PSA screening significantly reduces PCa mortality, and repeated screening may be of great significance in reducing PCa mortality in the population.
M. Luján Galán et al. (35)	15 years	The aim of the study was to assess whether PCa screening improves cancer-specific survival.	This study did not find a benefit of PSA screening in terms of overall survival and cancer-specific survival for PCa patients.
K. Talala et al. (36)	15 years	The study explored the effects of PSA screening on general and disease-specific health-related quality of life (HRQOL) in men with PCa.	At 15 years of follow-up, there were no significant differences in health-related quality of life between the PCa screening and control groups.
Z. Zhang (37).	—	This study was designed to evaluate the economics of early PSA screening for high-risk prostate cancer.	Compared to not screening, early prostate cancer screenings are more cost-effective for high-risk patients.
T. Pakarainen et al. (38)	17 years	This study investigated the effects of PSA screening on the incidence of PCa in different risk groups.	Screening for more than 2 cycles reduces the risk of advanced PCa, and this study highlights that repeated screening is necessary to realize screening benefits.
S. D. Walter et al. (12)	18.6 years	The aim of this study was to estimate the overdiagnosis rate of PSA screening.	This study confirms that there is some overdiagnosis of PSA screening, but the extent is uncertain.
C. Arsov et al. (39)	—	This study established the effectiveness of risk-adaptive PSA screening.	The prevalence of aggressive PCa screened for 45-year-old men is low.
M. Frånlund et al. (40)	22 years	The study examined the impact of PSA screening on PCa mortality.	PSA screening can significantly reduce the mortality of PCa.

The European Randomized Study of PCa Screening (ERSPC), the largest current study of randomized screening for PCa, found that PSA screening resulted in a significant reduction in PCa mortality after 9 and 11 years of follow-up (21). Due to the variation in the occurrence of PCa across different regions, the treatment methods for it also exhibit substantial differences among these regions. Therefore, the researchers also used the above data to carry out research analysis in each region. J. Hugosson et al. (34) used data from eight European countries in the ERSPC trial to determine whether PSA screening reduces PCa mortality up to 16 years. The results of the study that included 182,160 men confirmed that early PSA screening significantly reduced PCa mortality, and the absolute benefit was greater with longer follow-up. This study also confirms that repeated screening may be important in reducing PCa mortality in the population. M. J. Roobol et al. (24) evaluated PCa-specific mortality data from the Rotterdam portion of the ERSPC trial. 42,376 men aged 54 to 74 years were randomly assigned to screening and control groups at a frequency of 1 screening every 4 years. After a median follow-up of 12.8 years, PSA-based systematic screening reduced prostate-specific mortality by 32% in the 55-69 year age range. Besides, in order to explore the effects of PCa screening on different age stages, Okhorst et al. (25) used part of the Rotterdam data(34,833 men aged 55-69 years) from the ERSPC trial to explore the impact of PCa screening on mortality. The primary endpoint was PCa specific mortality. The results of the study found that PCa screening at the Rotterdam section of the ERSPC reduced the risk of PCa death by 51% compared to men who were not screened for PCa. In addition, J. Hugosson et al. (32) randomly assigned 20,000 men aged 50-64 years from the Goteborg population Registry to receive PSA screening and controls. Men in the screening group were invited to undergo PSA testing every two years until the median age of 69. The study followed for 18 years and found that systematic PSA screening showed a greater PCa mortality benefit among men who began screening at ages 55-59. Recently, M. Franlund et al. (40) analyzed the results of the 22-year follow-up of the Goteborg Randomized PCa screening trial. The results showed that the 22-year cumulative PCa mortality was 1.55% in the experimental group and 2.13% in the control group. These studies suggest that PSA-based screening can significantly reduce PCa mortality.

The researchers also attempted to explore the specific mechanisms by which PSA screening leads to reduced mortality in patients. C. Buzzoni et al. (28) evaluated the incidence of PCa in study groups according to the risk category at diagnosis to assess the potential impact on PCa mortality. Information on patient groups, centers, T and M stages, Gleason scores, serum PSA at diagnosis, age at randomization, duration of follow-up, and survival status were extracted from the ERSPC database. The results confirmed that in the screening group, the metastatic disease at diagnosis reduced markedly. The results of this study suggest that the reduction in metastatic disease during PSA screening is a major determinant of the reduction in PCa mortality. In addition, S. Neupane et al. (41) identified prognostic factors for patients dying from PCa in the Finnish PCa screening trial. The 15-year survival rate was significantly lower in the control group than in the screening group. The study showed that PSA screening led to earlier treatment of cases in the screening group.

However, other similar studies have not found a benefit from PSA screening in reducing mortality in men with PCa. M. Luján et al. (26) reported the long-term results of a PCa screening trial conducted in the Mediterranean region. A total of 4,276 men aged 45 to 70 years were randomly assigned to screening and control groups. After 15 years of follow-up, the study failed to demonstrate a benefit of PCa screening in terms of all-cause and PCa-specific mortality. M. Lujan et al. (30) also studied all-cause mortality and cancer-specific mortality in the Spanish branch of the ERSPC study. A total of 18,612 men aged 45 to 70 years were randomly assigned to the screening or control group for a median follow-up of 15.8 years. The study found no difference in cancer-specific mortality between the two groups. The lower long-term PCa mortality rates found in the above study may be the most important factor contributing to these results. Subsequently, M. Lujan Galan et al. (35) provided the latest results of the ERSPC Spanish Research Center follow-up after 21 years. No benefit of PCa screening in terms of overall survival or cancer-specific survival was found in the Spanish study portion of the 21-year follow-up. Similarly, R. M. Martin et al. (33) evaluated the effect of a single PSA screening intervention and standardized diagnostic pathways on PCa specific mortality. The Cluster Randomized Trial of PSA Testing for PCa enrolled 419 582 men aged 50 to 69 years. In clinical practices randomized to receive a single PSA screening intervention, there was no significant difference in PCa mortality after a median follow-up of 10 years compared to standard practice without screening. T. P. Kilpelainen et al. (23) evaluated mortality outcomes from the Finnish PCa screening trial, the largest component of the ERSPC. The results found that a relatively conservative screening regimen resulted in a small but not statistically significant reduction in PCa-specific mortality at 12 years, at the cost of moderate overdiagnosis. Furthermore, K.Talala et al. (36) compared general health-related quality of life (HRQOL) and disease-specific HRQOL in PCa patients with up to 15 years of follow-up in the Finnish population-based Randomized Study of PCa Screening. At 5 to 15 years of follow-up, there were no significant differences in health-related quality of life between the PCa screening and control groups.

#### 3.3.2 PSA screening and overdiagnosis of PCa

Studies have shown that organized screening reduces PCa mortality, but the effects of opportunistic screening have been largely unknown. R. Arnsrud Godtman et al. (27) compared the ability of organized and opportunistic screening to reduce PCa mortality and the risk of overdiagnosis. The Goteborg Screening study has randomly selected 10,000 men since 1995 for a PSA test every 2 years and recommends prostate biopsies for men with PSA≥2.5 ng/ml. The study found that organized screening reduced PCa mortality, but was associated with overdiagnosis. Opportunistic PSA testing had little impact on PCa mortality and led to more overdiagnosis. Besides, S. D. Walter et al. (12) estimated overdiagnosis rates using Finnish data from the ERSPC trial. The study defined the overdiagnosis rate as the relative excess cumulative incidence in the screening group at this time. Studies have shown some overdiagnosis in screening, but the extent is uncertain. In addition, a study from China suggests that early screening is more cost-effective than no screening for high-risk prostate cancer patients (37). Therefore, promoting early screening for high-risk prostate cancer patients is also a valuable strategy.

PCa screening relies on a careful balance of benefits in reducing PCa mortality and harms in terms of overdiagnosis and overtreatment. A. J. Vickers et al. (16) evaluated the impact of limiting PSA testing based on age and baseline PSA on overdiagnosis. Two independent cohorts (1,577 and 1,197 participants, respectively) were included in the PSA screening group, with a Swiss cohort that had not received PSA screening serving as a control group, and the included cohorts were followed for 25 years. Studies have found that overdiagnosis of PCa is closely related to age and PSA level. Limiting screening of men older than 60 to those with PSA above the median (>1 ng/ml) would critically reduce overdiagnosis. These studies suggest that in order to avoid overdiagnosis, PSA screening strategies need to be optimized, and screening populations need to be selected for appropriate age stages and PSA levels.

#### 3.3.3 Optimization of PSA screening strategy

Most of the available findings show a significant reduction in PCa mortality among men screened in the intervention group. Nevertheless, there are also studies that suggest it can lead to problems such as overdiagnosis. Therefore, it is necessary to optimize PSA screening strategies through existing studies in order to reduce PCa-specific mortality, overdiagnosis and cost, and improve the quality of life of patients.

The optimization of PSA screening strategy first needs to optimize the screening population. E. A. Heijnsdijk et al. (29) used a microsimulation model based on data from the ERSPC trial to predict the cost-effectiveness of various screening strategies starting at age 55 with a PSA threshold of 3. The study found that 2 to 3 PCa screenings in the 55-59 age group were cost-effective. Due to the loss of quality-adjusted life-years (QALYs) due to overdiagnosis, screening is less cost-effective in people older than 63 years. Similarly, A. Grenabo Bergdahl et al. (22) explored the risk of PCa after stopping screening. The study included 20,000 men with an average age of 69 years in the Goteborg area of Sweden who underwent PSA randomized screening. The sthdy found that nine years after PSA testing stopped, the incidence of potentially fatal cancers was comparable to that of men who had not been screened. This study reaffirms that PSA screening can be stopped in patients over 70 years of age. Besides, the researchers explored the appropriate age for PSA screening. S. Carlsson et al. (31) evaluated the effect of PSA screening initiated at ages 50 to 54 on PCa mortality. The study found that PSA screening for PCa reduces PCa mortality in men aged 50-54 years, which is comparable to the results of a previously reported randomized study of PCa screening in men aged 55-69 years in Europe in a similar follow-up. Guidelines may consider whether PSA screening guidelines recommend starting screening no later than age 50-54. A randomized trial (PROBASE) conducted by C. Arsov et al. (39) recruited 46 642 men aged 45 years to determine the efficacy of risk-adapted PSA screening starting at age 45 or 50 years. The prevalence of screen-detected aggressive PCa was very low among men aged 45 years. Therefore, PSA screening is recommended to start after 50 years old. At present, there is no accepted initial age for PCa screening.

Furthermore, the selection of the optimal PSA screening frequency is also particularly important for PCa screening. T. Pakarainen et al. (38) explored the effects of participation in screening on the incidence of PCa in different risk groups. Participants in the Finnish trial screening group (31,867 men) were classified based on screening frequency. The results showed that the incidence of low risk PCa increased with the number of screening times, while the incidence of intermediate and high risk PCa was not significantly associated with the number of screening times. Single screening shows no benefit in PCa incidence, and repeated screening is necessary to realize the screening advantage. E. Kovac et al. (18) assessed the long-term risk of any PCa and clinically significant PCa in men aged 55-60 years based on baseline PSA levels. The results found that baseline PSA levels in men aged 55 to 60 years were associated with a long-term risk of clinically significant PCa. These findings suggest that the frequency of repeat screening can be reduced in men aged 55-60 years with low baseline PSA levels (less than 2.00 ng/mL), and screening may be discontinued in men with baseline PSA levels below 1.00 ng/mL. To optimize the frequency of PSA screening, R. Landy et al. (42) assessed the 5 -, 10 -, and 15-year risks of invasive cancer and PCa-related death among men with baseline PSA levels of 0.5 ng per milliliter or less, 1 ng per milliliter or less, and 1.01 to 2.5 ng per milliliter. The study found that for 45% of men with PSA ≤ 1 ng/mL, a 5-year screening interval may be appropriate. Men ≥65 years of age with PSA ≤ 0.5 ng/mL may consider stopping screening.

### 3.4 Assessment for the risk of bias

**Figure 3:**
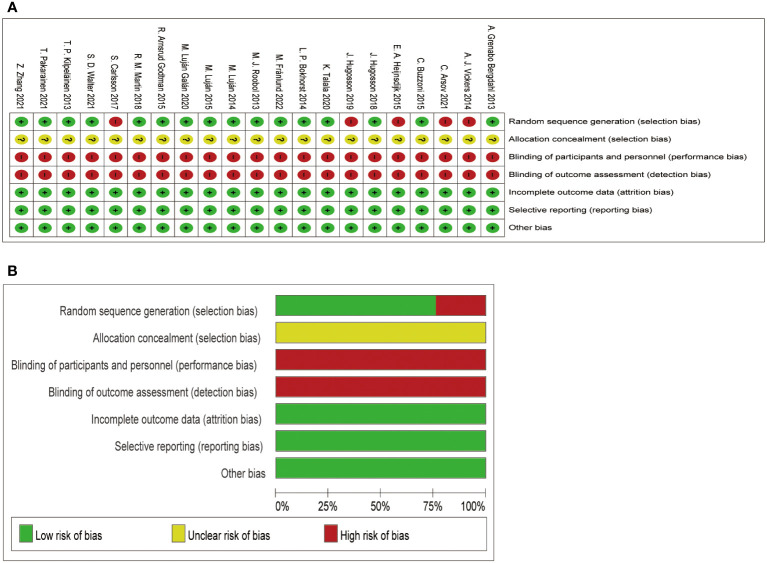
Assessment for the risk of bias. **(A)** shows the quality of the included literature evaluated by different items; **(B)** shows the proportion of quality assessment items in the included literature.

In the methodological assessment of the quality of the included literatures (**Figure 3**), 16 of them were at moderate risk of bias, and the quality was relatively high due to the large number of included study populations. In **Figure 3A**, those that meet the standard are “+” and those that fail to meet the standard are “-”. **Figure 3B** is a statistical chart of the proportion of each item in the methodological assessment.

## 4 Discussion

According to the 2001 updated guidelines from the American Cancer Society, there is still uncertainty regarding the overall effectiveness of PSA screening in reducing the likelihood of death from PCa. Numerous clinical trials investigating PSA screening have consistently demonstrated a decrease in mortality associated with PCa (24, 25, 28, 32, 34, 40, 41). Additional research revealed that the decline in the spread of cancer to other parts of the body as a result of PSA screening was the primary factor in the decrease of mortality caused by PCa. Moreover, PSA screening facilitated prompt intervention for individuals in the screening group. Nevertheless, certain randomized experiments have failed to demonstrate a survival advantage of PSA screening in males diagnosed with PCa (26, 30, 35, 43). With the rapid development of PCa treatment modalities, the long-term reduction of PCa mortality will weaken the impact of PSA screening on patient mortality, which may be the reason for the different results of PSA screening related studies.

Hence, the utilization of PSA screening for PCa is a subject of debate. PSA screening can effectively decrease patient-specific mortality in the diagnosis and treatment of PCa. However, some scholars argue that it may also increase the risk of overdiagnosis and overtreatment due to PCa’s slow-growing nature. Due to its comparatively sluggish progress, PSA screening is capable of identifying certain cancers that may otherwise go undetected during a man’s lifetime (44). However, it is important to note that diagnosing these abnormalities through screening does not effectively decrease mortality rates. The reason for this excessive diagnosis could be the existence of tumor slow-developing or inactive growths that can stay without symptoms for numerous years. Harmful consequences may arise as a result of screening in such instances. In addition, the above situation may also be due to regional differences, such as differences in the overall follow-up results of ERSPC study data and follow-up results in different regions (21, 24, 30, 34, 35). After all, the present therapeutic techniques for PCa are progressing swiftly, and the influence of varying degrees of treatment on the particular fatality rate of examined individuals is substantial.

Numerous studies have verified that the screening of PSA can enhance patients’ prognosis and decrease mortality rates. However, large-scale PSA screening not only leads to overtreatment but also imposes a certain economic burden on social health care. To address this issue, it is necessary to enhance the effectiveness of PSA screening strategies. Research has indicated that PSA testing for PCa can decrease PCa death rates in males between the ages of 50 and 54, and it is advised to initiate PSA screening by the age of 50 to 54. For patients at high risk of PCa, the age of screening should be appropriately reduced. Given that the predictive advantage of PSA screening for PCa diminishes in males above the age of 70, it is recommended to discontinue PSA screening in individuals aged 70 and above. Individuals between the ages of 55 and 59 should undergo two or three screenings for PCa. The incidence of PCa does not benefit from a single screening, and to achieve the advantage of screening, it is necessary to undergo repeated screenings. Furthermore, men with a PSA level of ≤1 ng/mL may find a 5-year screening interval suitable, and for men aged ≥65 with a PSA level of ≤0.5 ng/mL, the option of discontinuing screening could be contemplated. Furthermore, screening can be less frequent for men aged 55-60 years with a baseline PSA level below 2.00 ng/mL.

This study also has limitations, as most of the included studies had a risk of bias score of moderate risk of bias. In addition, a part of the included studies analyzed the data of multi-center studies in different regions or with different follow-up times, resulting in heterogeneity such as regional differences and different follow-up times in the included studies, so no Meta-analysis was performed. However, the population size of the selected studies is large, so the conclusions obtained through comprehensive analysis are also reliable.

## 5 Conclusions

To summarize, the aforementioned studies indicate that PSA screening is effective in reducing mortality specifically related to PCa. The overdiagnosis and overtreatment of PCa occur because of the low long-term specific mortality of PCa, which is due to the inert nature of PCa and advancements in comprehensive treatment technology. Hence, it is crucial to enhance the suitability of PSA screening for specific age groups, modify the screening frequency, and determine the optimal PSA levels. This will aid in the development of a personalized screening program, thereby enhancing the effectiveness of PSA screening in diagnosing PCa.

## Data availability statement

The original contributions presented in the study are included in the article/supplementary material. Further inquiries can be directed to the corresponding author.

## Author contributions

ZZ: Conceptualization, Data curation, Writing – original draft, Writing – review & editing. AT: Conceptualization, Investigation, Methodology, Writing – review & editing. JC: Data curation, Formal analysis, Investigation, Methodology, Validation, Writing – review & editing. YM: Data curation, Investigation, Methodology, Writing – review & editing. YuL: Data curation, Formal analysis, Investigation, Writing – review & editing. YaL: Data curation, Methodology, Writing – review & editing. YX: Data curation, Formal analysis, Funding acquisition, Investigation, Methodology, Supervision, Writing – review & editing.
